# Admission to intensive care can be reliably predicted using only clinical judgment

**DOI:** 10.1186/cc14622

**Published:** 2015-03-16

**Authors:** M Brabrand

**Affiliations:** 1 Hospital of South West Jutland, Esbjerg, Denmark

## Introduction

Not all patients in need of critical care arrive in clinical distress and some deteriorate after arrival. Identifying these patients early in their clinical course could potentially improve outcome. The present study was performed with the aim of assessing whether nursing and physician staff were able to identify patients in need of critical care using only clinical judgment and to compare this with the National Early Warning Score (NEWS).

## Methods

This was a prospective cohort study of all adult patients with a first-time admission to a medical admission unit at a 450-bed regional teaching hospital over a 3-month period in 2010. All subspecialties of internal medicine are present as well as a level 2 ICU. Upon first contact with the patient after arrival, nursing staff and physicians were asked to report their estimation of the probability of ICU admission (0 to 100%). Survival status was extracted from the Danish Civil Registry. All administrative details (including transfers to other hospitals, wards and ICUs) were extracted from the National Patient Registry, both ensuring complete follow-up. The discriminatory power (ability to identify patients at increased risk) was estimated using area under the receiver-operating characteristics curve. Calibration (accuracy) was assessed using Hosmer-Lemeshow goodness of fit test. Data will be reported as median (range) or proportions (95% confidence interval).

## Results

A total of 2,769 patients were included, median age 65 (18 to 100) years and 52% female. Thirty-day mortality was 4.5% and 2.2% were admitted to ICU. Median time to ICU admission was 1 (0 to 70) day. Nursing staff assessed 65% and physicians 26% of admissions. NEWS could be calculated for 85%. Nursing staff had a discriminatory power of 0.865 (0.786 to 0.944) with little variation with experience. Calibration was acceptable, except for the least experienced nurses (<5 years). Physicians had a discriminatory power of 0.789 (0.641 to 0.937), with little variation with experience. Calibration was very good, regardless of experience. NEWS had a discriminatory power of 0.809 (0.727 to 0.891) and poor calibration. There was no significant difference in discriminatory power between the three assessments.

## Conclusion

Both nursing staff and physicians were as good as NEWS at identifying patients at increased risk of ICU admission after admission to a medical admission unit. However, both nursing staff and physicians had better calibration (accuracy) than NEWS.

